# Patients’ perceptions of depression and coronary heart disease: a qualitative UPBEAT-UK study

**DOI:** 10.1186/1471-2296-14-38

**Published:** 2013-03-19

**Authors:** Rosemary L Simmonds, Andre Tylee, Paul Walters, Diana Rose

**Affiliations:** 1 Service User Research Enterprise (SURE), Institute of Psychiatry, King’s College London, London, UK; 2 Section of Primary Care Mental Health, Institute of Psychiatry, King’s College London, London, UK; 3 Health Services and Population Research Department, Institute of Psychiatry, King’s College London, De Crespigny Park, PO Box 34, London, SE5 8AF, UK

## Abstract

**Background:**

The prevalence of depression in people with coronary heart disease (CHD) is high but little is known about patients’ own perceptions and experiences of this. This study aimed to explore (i) primary care (PC) patients’ perceptions of links between their physical condition and mental health, (ii) their experiences of living with depression and CHD and (iii) their own self-help strategies and attitudes to current PC interventions for depression.

**Method:**

Qualitative study using consecutive sampling, in-depth interviews and thematic analysis using a process of constant comparison. 30 participants from the UPBEAT-UK cohort study, with CHD and symptoms of depression. All participants were registered on the General Practitioner (GP) primary care, coronary register.

**Results:**

A personal and social story of loss underpinned participants’ accounts of their lives, both before and after their experience of having CHD. This theme included two interrelated domains: interpersonal loss and loss centred upon health/control issues. Strong links were made between CHD and depression by men who felt emasculated by CHD. Weaker links were made by participants who had experienced distressing life events such as divorce and bereavement or were living with additional chronic health conditions (i.e. multimorbidity). Participants also felt ‘depressed’ by the ‘medicalisation’ of their lives, loneliness and the experience of ageing and ill health. Just under half the sample had consulted their GP about their low mood and participants were somewhat ambivalent about accessing primary care interventions for depression believing the GP would not be able to help them with complex health and social issues. Talking therapies and interventions providing the opportunity for social interaction, support and exercise, such as Cardiac Rehabilitation, were thought to be helpful whereas anti-depressants were not favoured.

**Conclusions:**

The experiences and needs of patients with CHD and depression are diverse and include psycho-social issues involving interpersonal and health/control losses. In view of the varying social and health needs of patients with CHD and depression the adoption of a holistic, case management approach to care is recommended together with personalised support providing the opportunity for patients to develop and achieve life and health goals, where appropriate.

## Background

The World Bank and the World Health Organization have predicted that coronary heart disease (CHD) and depressive disorder will be the two top causes of global health burden and disability by 2020 [1]. Estimates of the prevalence of major depression in patients with CHD range from 15% to 23% as compared to a general population prevalence of 4.6% [2,3]. Co-morbid CHD and depression is associated with a 2-fold increase in cardiac and all-cause mortality [4,5].

There has been increasing recent interest in multimorbidity and the bi-directional relationship between conditions such as coronary heart disease and depression in primary care [6]. Gunn and colleagues [7] have demonstrated from the Melbourne DIAMOND study, the increased prevalence of depression in a wide range of long term physical conditions in primary care. They found an increased likelihood of depression with increasing multimorbidity which appeared to be mediated by associated functional limitation and self rated health. Bhattarai and colleagues [8] also found an increasing prevalence of depression and physical comorbidity was associated with increased health care use and resource costs. Guthrie and colleagues [9] suggest integrating relevant single condition guidelines to make them more relevant for managing multimorbidity.

In contrast, recent qualitative work, using psychological models, has looked at the impact of multimorbidity on patient representations of their individual conditions as well as the representation of multimorbidity itself [10]. The authors concluded that such representations need to be incorporated into the design and delivery of multi-facetted interventions to modify health behaviours and to improve outcomes. Coventry and colleagues [11] have studied the barriers to managing depression in people with long term conditions in primary care and found that in partnership with patients, depression was often normalised by practitioners as an understandable reaction to physical illness. Furthermore, the current Quality and Outcomes Framework promoted a reductionist approach to case finding of people with depression and CHD. Once identified, practitioners can feel unsure how to frame discussions with patients around the topic of depression in coronary heart disease [12].

For the UPBEAT-UK qualitative study, reported on here, the aim was to explore patients’ own experiences, priorities and meaning making to inform the design of a nurse led personalised, case management intervention. Studies, such as COINCIDE [13] utilised psychological models to assess whether a collaborative care approach, using CBT based IAPT providers, could improve patient centred outcomes. In contrast the UPBEAT-UK study adopted an inductive, patient centred approach that could capture a broad range of concerns. We did not wish to employ existing psychological models or other theoretical approaches that may have limited the scope of the findings.

### Aims

This study aimed to explore (i) primary care (PC) patients’ perceptions of links between their physical condition and mental health, (ii) their experiences of living with depression and CHD and (iii) their own self-help strategies and attitudes to current PC interventions for depression.

### Ethical approval

Bexley and Greenwich ethics committee gave ethical permission for the study (reference 07/H0809/38), and approval was obtained from NHS trust research governance offices in South East and South West London. The Researcher (RS) consented the participants.

## Methods

### Sampling

The sampling frame was the UPBEAT-UK cohort study database of primary care patients with CHD. At the time of recruitment (December 2008 to January 2009) this numbered 376. Participants were drawn from GP, primary care CHD registers. Patients were recruited to the cohort study from four South London Boroughs (Lambeth, Lewisham, Southwark and Croydon) coterminous with Primary Care Trusts (PCTs) in South East and South West London, which all serve ethnically and socially diverse populations. Of the 376 participants in the cohort, 47 screened positive for depression using the PHQ2 [14]. Researchers from the main UPBEAT-UK study screened participants. At recruitment, participants were given the option of being interviewed in depth about their CHD and depression. 42 participants agreed. Of these, 5 were included in a pilot study to refine the interview topic guide. 1 of the remaining 37 declined to be interviewed and 3 could not be contacted or had died. Of the remaining 33, 30 were interviewed at which point interviewing was stopped as saturation had been reached.

### Data collection

One researcher (RS) conducted all the interviews, which were digitally recorded, transcribed verbatim and entered into NVivo8 qualitative software for analysis and data management. An interview topic guide was informed by the aims of the research, literature review, discussion with co-authors and findings from a preliminary, unstructured, interview study with depressed, cohort study participants. The topic guide covered perceptions of the relation between their physical and mental health, their experiences of living with depression, their opinions about interventions and their own self-management strategies. Patients were interviewed by the first author (RS) in a private space, either in their own homes or at their GP surgery.

### Analysis

Interviews were analysed using a thematic approach [15,16] involving a process of constant comparison between cases, identifying patterns and qualities in the texts [17]. Texts were coded and re-coded iteratively until category saturation of each theme was achieved.

During the process of analysis, coding frames, themes and anomalous data were discussed regularly with a senior member of the research team (DR) and with members of the UPBEAT-UK research team, to corroborate findings.

## Results

### Participants

Table 1 shows the characteristics of participants.

**Table 1:** Characteristics of participants

		
**Gender**	Female	N = 11 37%
	Male	N = 19 63%
**Age**	Range	47-85 years
	Mean	65 years
	Median	63 years
**Ethnicity**	White British	70%
	African/Afro-Caribbean and Asian	30%
**Depression scores (PHQ**-**9)**	Range	8-27
	Mean	16.96
	Standard deviation	5.92
**Consulted GP for low mood**	Female (n = 11)	36%
	Male (n = 19)	42%

### Thematic analysis

‘Loss’ was a theme that underpinned all of the interviews in this study. Some participants made direct links between CHD and their psychological state, whilst others made more indirect links attributing their feelings to interpersonal, social and health related factors.

**Figure 1:**
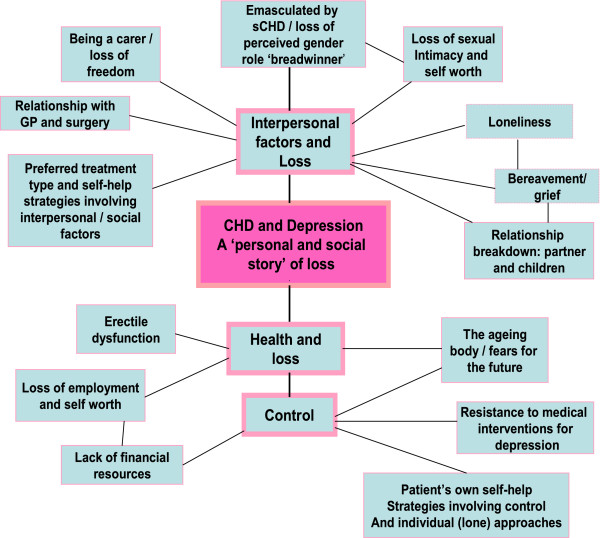
A model of participants’ perceptions of links between coronary heart disease and depression.

Not all of the themes, outlined in Figure 1, are included in this paper. The following table outlines key topics and associated themes that have been included (Table 2).

**Table 2:** Key topics and themes identified in the interviews

**Topics**	**Themes**
1. Participants’ perceptions of links between CHD and depression	Strong links
	• Emasculated by CHD
	• No longer the breadwinner
	• Erectile dysfunction
	Weaker links
	• Relationship breakdown
	• Bereavement
	Weak links
	• Depressed because of multiple health conditions
	• The experience of ageing
2. Participants’ perceptions of primary care interventions for depression	Interventions participants found helpful
	• Talking therapies
	• Exercise and rehabilitation
	Interventions participants did not find helpful
	Anti-depressants
3. Participants’ own self-help strategies	Self talk and thinking strategies
	• Counting blessings
	Meditation and yoga

### Direct links identified

#### Emasculated by CHD

Half of the male participants explicitly stated a link between CHD and depression. The way men described their feelings was closely connected to a perceived loss of manliness in relation to ideas of traditional masculinity. The theme of emasculation included loss of role as ‘breadwinner’ and the impact of suffering from erectile dysfunction.

#### No longer the ‘breadwinner’

In the following two extracts participants explained how having a major heart attack had affected them:

“…when it first happened [heart attack] it’s like someone’s cutting your balls off to be honest. Like your manhood or whatever, I don’t know…feeling of uselessness I suppose because I’ve always been like, lets say, taking the bacon home.” (Participant 1284, male).

“…it’s going back to the role of being a breadwinner I would say…money was getting desperate, things were getting really bad and I kept blaming myself cos I couldn’t do it and I felt absolutely useless, I couldn’t do nothing to help out…” (Participant 1236, male)

Loss of employment and the traditional male gender role of ‘breadwinner’ was perceived by male participants as a loss of masculine identity and self-esteem.

#### Erectile dysfunction

The following participant explained how experiencing erectile dysfunction had affected his sense of self worth.

“…just loads of things build up – you don’t feel like a man and then the worst thing of all is when you don’t feel like a man ‘downstairs’. If I was fifty six I wouldn’t worry about it but I’m forty six so there’s the difference. I think to meself, ‘no I am still young enough to..’ and its another reason why I don’t like…I still want to go out, I’ve still got me charm and me confidence, I always had that cheeky boy sort of thing but now I’m scared to get into a relationship because I can’t perform. So what is the point, I think ‘what’s the point?’ And then I get depressed.” (Participant 1060, male)

This extract shows the profound impact that erectile dysfunction and CHD can have on a man – to his sense of self worth, emotional/social well-being and mental health.

#### Indirect links identified

In this category participants did not make specific causal links between CHD and depression. Instead, participants cited issues of partnership breakdown and bereavement as the factors that had contributed to their physical and psychological states.

#### Relationship breakdown

Fifty per cent of participants reported having been very stressed and unhappy in their lives due to relationship breakdowns and divorce. Participants partly attributed their feelings of distress to these experiences and partly to their physical condition.

“I don’t want to sound like an actress on wheels but um, my heart is broken and it manifests itself physically as well as mentally – you have a heart *attack!”* (Participant 1049, female)

This participant used the metaphor of a broken heart to connect her experience of marriage breakdown with having a heart attack and depression.

#### Already depressed by negative life events

The following participant described how a number of serious life events and losses had affected him:

“I was pretty devastated with the boy dying. Me and my wife had split up, which I’d know, speaking to people who it had happened to, that it either brings you closer together, the death of a child, or the other way. Unfortunately, mine went the other way although the marriage was a bit iffy, being a lorry driver and things like that…I think just devastation really because I was jobless, homeless and financially more or less skint.” (Participant 1284, male)

For some participants stressful life events can have a ‘domino’ effect with one traumatic event (death of a stepson) tipping the balance in other spheres of life and taking their toll on physical and mental health.

#### Bereavement

The loss of a child or death of a partner were issues that had also caused a great deal of distress in the lives of participants.

The following participant was feeling very sad and lonely after the death of his wife:

“She died just 5 years ago, she was buried on her birthday…she would have been 69 then and I met her at 16, so that’s a long, long time…You think of them, sometimes you think I wish she was here, especially when you want a cuddle of a night, I think ‘oh I wish she was here’…Yeah, huge loss, I think that was one of the things that contributed to me having problems with the old ticker because you worry so much…” (Participant 1321, male)

Some of the bereaved participants in this study believed that there was a causal link between experiencing grief and heart disease.

### Weak links identified

#### Depressed because of multiple health conditions

Participants living with multiple, chronic health conditions reported a rather weak association between CHD and depression. These participants attributed feeling ‘low’ to being in pain or living a life curtailed by physical illnesses.

The following participant was 52 years old and suffering from a number of health conditions that were dominating his life:

“Well, I’ve got diabetes, which I take insulin for and I’ve got high blood pressure and I suffer from chest problems…The thing is they treated me for asthma but now they’ve decided it’s not that, it’s gosh I forget what it is, some kind of breathing problem. So that’s some of the other reasons and of course I’m overweight and those things don’t help so I get told off by the diabetic clinic and heart clinic…I should be losing weight but it’s a vicious circle. I have an underactive thyroid and take a lot of thyroxine and that has to be measured and I just find it very difficult to lose weight.” (Participant 1081, male)

This participant’s account illustrates how various health conditions, including depression, can impact upon each other until the patient feels caught and trapped within a cycle of poor health, stress and unhealthy coping mechanisms.

#### The experience of ageing

Just over one third of the sample in this study was aged between 70 and 85 years. For some of these participants the experience of ageing and ill health was also connected to feeling low.

Older participants were not only struggling to come to terms with the physical, social and economic implications of ageing and ill health but the fear of cognitive deterioration as well:

“That’s what frightens me, if I get to the point of when I have more senior moments, you know, forgetting names, that sort of things, going a bit gaga and that frightens me because the kids always go ‘it’s all right Mum, we will put you in a home in Australia so we won’t have to visit you!’ but that does worry me, what’s going to happen in the next few years if you do deteriorate, ie the walking. You are more dependent on people to do things for you, that worries me, I don’t know how I am going to cope with that, I don’t want them to look after me, why should they? They have got their own lives but I don’t want to go in a home but what do I do cos I can’t live on my own. That worries me…!” (Participant 1189, female)

This extract illustrates how the experience of ageing can include anxiety about the future, about losing mobility, mental acuity, becoming dependent and the loss of agency and control.

Loneliness was also an issue for some older participants and the impact of being alone and experiencing the ageing process was explained by the following participant:

“…sometimes you would like another person sitting in the armchair, sometimes you do get lonely don’t you, on your own? I mean, you can be lonely in a room full of people but sometimes when you are sitting indoors you think ‘oh I wish there was someone living here with me, just to have a chat now…Sometimes I do think I wish I had someone here, a partner or something like that…but it does frighten me getting older, cos I don’t know what’s going to happen.” (Participant 1189, female)

Participants who have lost partners through death or divorce might find the experience of aging and ill health a lonely and uncertain process.

### Participants’ perceptions of primary care interventions for depression

#### Interventions for depression participants found helpful

Participants found talking therapies, cardiac rehabilitation programmes and supervised exercise helpful.

#### Talking therapies

“I would like to be able to open myself to one person who understands me, my mentality, my way of thinking and then they can give me advice, I mean, we need communication, without we can do nothing.” (Participant 1313, male)

Participants seemed to prefer interventions for CHD and depression that included either or both of the following: interpersonal talking approaches and the opportunity for social interaction and support.

Other services that had been offered to patients and were considered helpful included: cardiac rehabilitation and supervised exercise.

#### Cardiac rehabilitation programmes

Most participants had attended a 6 week cardiac rehabilitation (CR) programme post MI. Everyone who attended CR found this a helpful and positive experience as illustrated by the following comments:

“I thought it was tremendous…very useful indeed” (Participant 1049, female); “…it was a laugh” Participant 1060, male; “It’s quite good, we start slowly, slowly but they tell you what to do, they don’t force you…very helpful” (Participant 1300, male). “…they get on the chairs, form a circle and just…you know, you’re having a chat for little while and then do your exercises, so it’s all friendly.” (Participant 1305, female). “…everybody started encouraging you and you put in an effort to do it.” (Participant 1251, male)

Participants found CR programmes to be useful and enjoyable because they helped them to regain confidence and develop a sense of mastery over symptoms. Participants also enjoyed the social support and encouragement they received from other group members. Most participants would have liked to have continued attending a similar supervised, CHD dedicated, programme of exercise.

#### Supervised exercise

Some participants were given a prescription for exercise by their GP which enabled them to access fitness centres. Some fitness centres ran specific groups for people with CHD however, there appeared to be variance between levels of supervision at the different fitness centres. Interviewees generally preferred higher levels of supervision and a group environment.

### Interventions for depression that patients did not find helpful

#### Antidepressants

Although nearly one third of the sample had taken antidepressants or were still taking them, only two of these participants reported any improvements or benefits. In addition, some of these participants had experienced incompatibility problems between anti-depressants and drugs for CHD or unpleasant side-effects.

The remaining two-thirds of the sample said they would not consider taking anti-depressants because: they were already taking a lot of medication; felt that they were being ‘fobbed off’ by their GP and through hearing about the negative experiences of family and friends.

The following extract illustrates how being prescribed antidepressants is perceived by some patients:

*Researcher:* “*So* what you have been saying really that if you felt low you wouldn’t be too interested in coming and talking to your doctor about it?”

*Participant:* “No”

*Researcher:* “Is it partly that you feel you are burdening them?”

*Participant:* “No, not so much you are burdening, they have heard it all before, you are there for 15 minutes and you are out the door with a pill. No!” (Participant 1189, female)

This participant expressed some cynicism about what a GP can do to help someone who is experiencing a number of social problems as well as poor health.

### Participants’ own self-help strategies

Although some participants were reluctant to consult their GPs about feelings of distress or depression, most participants had used some coping and self-management strategies.

#### Self talk and thinking strategies

Some participants talked about having ‘down days’ and interviewees described the thinking strategies they used to cope with these occasions:

*Participant*: Well, normally, I just try and sort of talk to meself and say ‘come on you’ve got to start doing something’ and you know, normally the mood will lift. (Participant 1081, male)

In contrast other participants found that ‘counting their blessings’ helped them when they were feeling low:

*Participant*: “When you see the people outside they are worse than me, if you go to the hospital some people can’t eat, all that thing you know, even they don’t know where they are, I am still better off in that way…That’s what keeps me going you know.” (Participant 1313, male)

Participants preferred to adopt a stoical approach to the problems in their lives and although this helped them to see their situation in a more positive light it also deterred them from seeking help.

#### Meditation and yoga

Some participants found that practising meditation and/or yoga was a helpful strategy to calm thoughts, emotions and alleviate physical discomfort:

…I lie in bed and I have an attempt at meditation, she [mental health professional] taught me and I have to do it now before I go to sleep I have 10 minutes meditating…She used to sit me there for an hour and we would get on with this and I used to think ‘this is a load of rubbish’ and then I found it was calming. It made me think of good things, it made me think of life, it got me out of thinking of the bad things and I still do it now. (Participant 1236, male)

Participants found meditation techniques and yoga breathing exercises helpful and used them when they felt anxious or were experiencing feelings of panic.

## Discussion

Many of the themes found in this study are common to older populations with depression and not specific to those with coronary heart disease e.g. loss and grief, social isolation, medical illness and disability [18,19]. However, there were also themes identified that are specific to this population. Men who felt emasculated by CHD made the strongest connection between CHD and depression, directly attributing the latter to the former. For other participants the link between CHD and depression was more tenuous. Some had experienced a number of adverse life events, such as partnership breakdown that had caused them distress before or concurrent with being given a diagnosis of CHD. Participants who felt lonely and isolated reported a weaker link between CHD and depression because their loneliness itself made them depressed. The experience of aging and ill health again was experienced by some as an uncertain and lonely process. Participants expressed ambivalent attitudes to seeking primary care help for depression. Some participants thought their GP would not be able to help them with psycho-social problems and did not want to be ‘fobbed off’ with a ‘pill’. Most of the participants in this study had attended cardiac rehabilitation and enjoyed the peer support and re-building of bodily confidence in a safe environment. Whilst this is not a new finding, the level of unanimity amongst participants is striking. Participants valued talking therapies but none mentioned specific forms of talking therapy beyond ‘counselling’. What they wanted was ‘someone to talk to’. The references to yoga and meditation, on the other hand, suggest that new psychological approaches such as mindfulness might be appreciated.

This study is part of the UPBEAT UK project which aims to design a nurse-led, primary care intervention for patients with comorbid CHD and depression. The results of this qualitative study have informed the intervention so making it reflective of patients’ views and wishes.

### Strengths and limitations

The methodological strength of the study was the use of in-depth interviews that allowed participants to raise a diverse range of issues that mattered to them, within the context of living with CHD and depression. The strong links made between CHD and depression by men who felt emasculated by CHD was an interesting and original finding. Our study however had some limitations. This was a small study (N = 30) and all interviews were conducted with one researcher. To avoid over-subjective interpretations of the data, the content of interviews was discussed at all stages with a senior member of the research team (DR), who has particular expertise in conducting research on service user perspectives, and with members of the UPBEAT-UK research team. During the coding of interviews, a grounded approach was used so that themes were generated from the data and participants own words were used to label the codes. This process helped to keep speakers’ words in context thereby avoiding any misrepresentation of their intended meaning in the analysis. The nature of the research questions and remit of the study meant that some data was not included in the final analysis; by focusing on patients’ perceptions of physical illness and mental distress this study has necessarily produced a partial picture. For example, data relating to participants’ descriptions of the enjoyable aspects of their lives was not included, unless considered directly relevant to the remit of the study. A limitation in the design of this study was the absence of the views and experiences of significant others of patients with CHD and depression. Research suggests that families and spouses need help to manage the emotional turmoil of a serious cardiac event and that support to significant others and inclusion in care planning can improve outcomes in patients [20-23]. A further limitation is that the study was conducted in a metropolitan area. Although not all parts of this area were deprived a substantial portion was and so there may be difficulties in generalising the findings to more affluent parts of the UK.

It is apparent that some of our participants experienced multi-morbidities (see also Background). However, we did not sample for these nor identify them clinically so they are apparent as a theme in the participants’ discourse but we cannot be explicit about their contribution to depression in any quantitative way. That is not the aim of a qualitative study although it would be interesting to know if the risk of depression increases with multi-morbidities as has been found in epidemiological studies [7].

A further limitation of our study is that we have not distinguished in our interviews between new onset depression post MI which has been found to affect cardiac outcome [24] and other depression because we have recruited from a heterogenous group of patients who may have been on the CHD registers for many years after an MI or who may have evidence of CHD without having had an MI.

## Conclusion

This study formulated the links that patients make between their CHD and depression in their own words. The findings influenced the personalised, nurse-led service that the UPEAT-UK trial then investigated. Other qualitative investigations [13] have used model-driven designs whereas this is the first study to ground an understanding of patients’ views in their own terms and in their own language.

## Competing interests

The authors declare that they have no competing interests

## Authors’ contributions

DR, AT and PW conceived the study. RS conducted the interviews and the analysis. RS and DR discussed the results. RS wrote the first draft of the manuscript. DR, AT and PW all reviewed the manuscript and were involved in its critical revision before submission. All authors read and approved the final manuscript.

## Authors’ information

The UPBEAT-UK Research Team consists of: Andre Tylee (PI), Mark Ashworth, Elizabeth Barley, June Brown, John Chambers, Anne Farmer, Zoe Fortune, Mark Haddad, Sally Hampshire, Morven Leese, Anthony Mann, Paul McCrone, Anita Mehay, Joanna Murray, Diana Rose, Gill Rowlands, Rosemary Simmonds, Alison Smith, Paul Walters, John Weinman.

## Pre-publication history

The pre-publication history for this paper can be accessed here:

http://www.biomedcentral.com/1471-2296/14/38/prepub

## References

[B1] Murray CJ Lopez AD Alternative projections of mortality and disability by cause 1990–2020: Global burden of disease study Lancet 1997 349 9064 1498 1504 10.1016/S0140-6736(96)07492-2 9167458

[B2] Brotman DJ Golden SH Wittstein IS The cardiovascular toll of stress Lancet 2007 370 1089 1100 10.1016/S0140-6736(07)61305-1 17822755

[B3] Grace SL Abbey SE Ruxandra P Shnek ZM Irvine J Stewart DE Longitudinal course of depressive symptomatology after a cardiac event: Effects of gender and cardiac rehabilitation Psychosom Med 2005 67 52 58 10.1097/01.psy.0000151486.28349.70 15673624 PMC2928242

[B4] Dickens CM McGowan L Percival C Tomenson B Cotter L Heagerty A Creed FH Contribution of distress and anxiety to impaired health-related quality of life following first myocardial infarction Br J Psychiatry 2006 189 367 372 10.1192/bjp.bp.105.018234 17012661

[B5] Turvey CL Klein DM Pies C Distress, physical impairment and treatment of distress in chronic heart failure J Cardiovascular Nursing 2006 21 3 178 185 10.1097/00005082-200605000-00005 16699357

[B6] Mercer S Mamaging patients with mental and physical multimorbidity BMJ 2012 345 e5559 10.1136/bmj.e5559 22945951

[B7] Gunn J Ayton DR Densley K Pallant JF Chondros P Herrman HE Dowrick CF The association between chronic illness, multimorbidity and depressive symptoms in an Australian primary care cohort Soc Psychiatr Psychiatr Epidem 2012 47 2 175 184 10.1007/s00127-010-0330-z 21184214

[B8] Bhattarai N Charlton J Rudisill C Gulliford MC Prevalence of depression and utilization of health care in single and multiple morbidity: a population-based cohort study Psychol Med 2012 1 1 9 epub 23114010 10.1017/S0033291712002498

[B9] Guthrie B Payne K Alderson P McMurdo MET Mercer S Adapting clinical guidelines to take account of multimorbidity BMJ 2012 345 e6341 10.1136/bmj.e6341 23036829

[B10] Bower P Harkness E Macdonald W Coventry P Bundy C Moss-Morris R Illness representations in patients with multimorbid long-term conditions: Qualitative study Psychol Health 2012 27 10 1211 1226 10.1080/08870446.2012.662973 22390140

[B11] Coventry PA Hays R Dickens C Bundy C Garrett C Cherrington A Chew-Graham C Talking about depression: a qualitative study of barriers to managing depression in people with long term conditions in primary care BMC Fam Pract 2011 Mar 22;12;10 10.1186/1471-2296-12-10 PMC3070666 21426542

[B12] Barley EA Murray J Walters P Tylee A General practitioners’ and practice nurses’ views and experiences of distress and depression in coronary heart disease: Qualitative study BMJ 2010 1 1 10.1186/1471-2296-13-1 PMC3276431 22221509

[B13] Coventry PA Lovell K Dickens C Bower P Chew-Graham C Cherrington A Garrett C Gibbons CJ Baguley C Roughley K Adeyemi I Keyworth C Waheed W Hann M Davies L Jeeva F Roberts C Knowles S Gask L Collaborative Interventions for Circulation and Depression (COINCIDE): study protocol for a cluster randomised controlled trial of collaborative care for depression in people with diabetes and/or coronary heart disease Trials 2012 13 139 10.1186/1745-6215-13-139 22906179 PMC3519809

[B14] Kroenke K Spitzer RL Williams JB The PHQ-9: validity of a brief distress severity measure J Gen Internal Medicine 2001 16 9 606 13 10.1046/j.1525-1497.2001.016009606.x PMC1495268 11556941

[B15] Flick UE Von Kardorff I A companion to Qualitative Research 2004 London: Sage

[B16] Miles M Huberman M Qualitative Data Analysis: an expanded sourcebook 1994 Beverly Hills: Sage

[B17] Glaser BG Strauss AL The discovery of grounded theory: strategies for qualitative research 1967 Chicago: Aldine

[B18] Roberts RE Kaplan GA Shema SJ Strawbridge WJ Does growing old increase the risk for depression? Am J Psychiatr 1997 154 10 1384 1390 9326820 10.1176/ajp.154.10.1384

[B19] Lawrence V Murray J Banerjee S Turner S Sangha K Byng R Bhugra D Huxley P Tylee A Macdonald A Concepts and causation of depression: A cross-cultural study of the beliefs of older adults Gerontologist 2006 46 1 23 32 10.1093/geront/46.1.23 16452281

[B20] Kristofferzon M Lofmark R Carlsson M Coping, social support and quality of life over time after myocardial infarction J Adv Nursing 2005 52 2 113 24 10.1111/j.1365-2648.2005.03571.x 16164472

[B21] Moser DK Dracup K Role of spousal anxiety and depression in patients’ psychosocial recovery after a cardiac event Psychosom Med 2004 66 527 32 10.1097/01.psy.0000130493.80576.0c 15272098

[B22] Macdonald W Mead N Bower P Richards D Lovell K A qualitative study of patients’ perceptions of a minimal psychological therapy Int J of Soc Psychiatr 2007 53 23 35 10.1177/0020764006066841 17333949

[B23] Condon C McCarthy G Lifestyle changes following acute myocardial infarction: Patients perspectives Eur J Cardio Nursing 2006 5 37 44 10.1016/j.ejcnurse.2005.06.005 16055382

[B24] Dickens C McGowan L Percival C Tomenson B Cotter L Heagerty A Creed F New onset depression following myocardial infarction predicts cardiac mortality Psychosomatic Med 2008 70 4 450 455 10.1097/PSY.0b013e31816a74de 18434496

